# Spousal Concordance of Cardiovascular Risk Factors in Newly Married Couples in China

**DOI:** 10.1001/jamanetworkopen.2021.40578

**Published:** 2021-12-22

**Authors:** Ravi Retnakaran, Shi Wu Wen, Hongzhuan Tan, Shujin Zhou, Chang Ye, Minxue Shen, Graeme N. Smith, Mark C. Walker

**Affiliations:** 1 Leadership Sinai Centre for Diabetes, Mount Sinai Hospital, Toronto, Ontario, Canada; 2 Division of Endocrinology, University of Toronto, Toronto, Ontario, Canada; 3 Lunenfeld-Tanenbaum Research Institute, Mount Sinai Hospital, Toronto, Ontario, Canada; 4 OMNI Research Group, Department of Obstetrics and Gynecology, University of Ottawa, Ottawa, Ontario, Canada; 5 Clinical Epidemiology Program, Ottawa Hospital Research Institute, Ottawa, Ontario, Canada; 6 Department of Epidemiology and Community Medicine, University of Ottawa, Ottawa, Ontario, Canada; 7 School of Public Health, Central South University, Changsha, China; 8 Liuyang Municipal Hospital of Maternal and Child Health, Beizheng, Liuyang, China; 9 Queen’s Perinatal Research Unit, Department of Obstetrics and Gynecology, Queen’s University, Kingston, Ontario, Canada

## Abstract

**Importance:**

Several studies have shown that older married couples share a propensity for accruing the same cardiovascular risk factors such as hypertension and dyslipidemia. However, it remains unclear if these spousal associations reflect their shared home environment and lifestyle or the tendency to choose a partner with a similar perspective on lifestyle choices and behaviors (assortative mating). Evaluating these associations in young, newly married couples may help to differentiate between these 2 possibilities.

**Objective:**

To evaluate the spousal concordance of cardiovascular risk factors in young, newly married couples.

**Design, Setting, and Participants:**

This prospective cohort study recruited 831 couples around the time of marriage registration in Liuyang, China, from February 1, 2009, to November 4, 2015. Statistical analysis was performed from April to May 2021.

**Main Outcomes and Measures:**

Spousal concordance of cardiovascular risk factors. Both partners underwent systematic assessment of cardiovascular risk factors, including evaluation of anthropometrics, blood pressure, and fasting lipids.

**Results:**

Among the 831 participating couples, mean (SD) age was 24 (3) years in the women and 26 (4) years in the men. There were significant correlations between spouses in systolic blood pressure (*r* = 0.43; *P* < .001), diastolic blood pressure (*r* = 0.36; *P* < .001), total cholesterol (*r* = 0.13; *P* < .001), low-density lipoprotein cholesterol (*r* = 0.11; *P* = .003), high-density lipoprotein (HDL) cholesterol (*r* = 0.22, *P* < .001), and triglycerides (*r* = 0.13; *P* = .001). After adjustment for covariates (age, household income, education level, smoking, and either body mass index or waist circumference), significant correlations persisted between spouses in their systolic blood pressure (*r* = 0.42; *P* < .001), diastolic blood pressure (*r* = 0.34; *P* < .001), HDL cholesterol (*r* = 0.17; *P* < .001), and triglycerides (*r* = 0.10; *P* = .04).

**Conclusions and Relevance:**

This cohort study found spousal concordance of cardiovascular risk factors among young newly married couples. Assortative mating based on these concordant risk factors at the time of marriage may partially explain the shared vascular risk profile of older marital partners and raises the possibility of couple-based care.

## Key Points

**Question:**

Are there spousal associations of cardiovascular risk factors in young, newly married couples?

**Findings:**

In this cohort study involving 831 newly married couples, there were significant correlations between spouses in systolic blood pressure, diastolic blood pressure, total cholesterol, low-density lipoprotein cholesterol, high-density lipoprotein cholesterol, and triglycerides.

**Meaning:**

This study’s finding of spousal concordance of cardiovascular risk factors in young, newly married couples suggests assortative mating (the tendency to choose a partner with similar perspective on lifestyle and/or behavior) may partially explain the shared cardiovascular risk factor profile that has been documented in older marital partners.

## Teaser

This cohort study evaluates whether there is spousal concordance of cardiovascular risk factors among young, newly married couples in Liuyang, China.

## Introduction

Both genetic susceptibility and environmental factors (such as lifestyle practices) may contribute to the development of cardiovascular disease (CVD). Accordingly, it is often challenging to quantify the precise contributions of genetic and environmental factors to the development of CVD. In this context, married couples can provide an interesting model wherein 2 individuals share a home environment and lifestyle but usually are not genetically related to one another. Indeed, with respect to lifestyle, there is intracouple concordance of behavioral factors such as diet, physical activity, and smoking.^1,2,3^ As such, it may not be surprising that many studies have shown that older married couples share a propensity for accruing the same cardiovascular risk factors such as hypertension and dyslipidemia.^1,2,3,4,5,6,7^ A meta-analysis of such studies revealed that the spouse of a patient with hypertension has 41% higher odds of also having hypertension, as compared with the partner of an unaffected individual.^7^ Moreover, given spousal concordance of vascular risk factors, marital partners share similar projected 10-year cardiovascular risk estimates, wherein approximately two-thirds of the vascular risk of an individual can be explained by the projected risk of their partner.^8^ Thus, married partners may share not only a life and a home but also cardiovascular health and disease.

Two main hypotheses for explaining the spousal concordance of cardiovascular risk factors are that the observed associations may reflect (1) the impact of the shared home environment and lifestyle or (2) the tendency to choose a partner with a similar perspective on lifestyle choices and behaviors (assortative mating).^1^ However, previous studies have found that it is difficult to determine the relative contributions of these 2 factors.^1^ In this context, we hypothesized that an approach to addressing this question would be to evaluate the spousal concordance of cardiovascular risk factors in young, newly married couples, in whom the potential effect of the shared home environment is likely yet to emerge.

## Methods

This study was conducted in the setting of a preconception cohort study, in which couples in the Liuyang region of Hunan province (China) were recruited at the time of marriage.^9,10^ In this region, couples attend a premarriage health clinic for assessment at the time of marriage registration. To establish this cohort, we recruited women from these clinics at Liuyang Maternal and Infant Hospital if they indicated an intention to conceive in the next 6 months. The partners of participating women were also invited to participate in the study. Both partners underwent baseline evaluation at recruitment and then, whenever they subsequently conceived, the women were followed across gestation to delivery. The current analysis was limited to the 831 couples in which both partners completed fasting bloodwork at baseline. The study was approved by the institutional research ethics boards of Central South University (Changsha, Hunan, China), Ottawa Hospital Research Institute (Ottawa, Canada), and Mount Sinai Hospital (Toronto, Canada), and all participants provided written informed consent. The study was conducted from February 1, 2009, to November 4, 2015. This study has been reported in accordance with the Strengthening the Reporting of Observational Studies in Epidemiology (STROBE) reporting guideline.

At the baseline visit, both partners completed interviewer-administered questionnaires and underwent physical examination by trained study staff, which included measurement of weight, height, waist circumference, and blood pressure. Blood pressure was measured in a seated position, after 10 minutes of rest, using an automated noninvasive blood pressure monitor, with 2 measurements performed 10 minutes apart and the mean recorded. Blood samples were drawn from both partners after overnight fast, enabling measurement of total cholesterol, high-density lipoprotein (HDL) cholesterol, and triglycerides by standard clinical biochemistry. Low-density lipoprotein (LDL) cholesterol was determined by Friedewald equation.

Adverse pregnancy outcomes that were noted included preterm delivery (delivery prior to 37 weeks), gestational diabetes and preeclampsia. Gestational diabetes was diagnosed on 2-hour 75-g oral glucose tolerance test if one of the following thresholds were met: fasting glucose greater than or equal to 5.1 mmol/L; 1-hour glucose greater than or equal to 10.0 mmol/L; or 2-hour glucose greater than or equal to 8.5 mmol/L. Preeclampsia was diagnosed based on blood pressure greater than or equal to 140/90 mm Hg and 24-hour urine protein greater than 0.3 g or positive random urine protein, at greater than or equal to 20 weeks gestation.

### Statistical Analysis

All analyses were performed using SAS version 9.4 (SAS Institute) from April to May 2021. Continuous variables were tested for normality of distribution, with natural log-transformation applied where necessary for skewed variables. Because it was unclear whether spousal associations of cardiovascular risk factors would be present for newly married couples, we applied a nonparametric method (Spearman correlation) to measure the degree of association between the risk factors in women and their partners. Spearman univariate correlations of cardiovascular risk factors were determined between women and their partners in the full study population and after stratification into (1) couples in which neither partner smoked and (2) couples in which at least 1 partner smoked. We next considered factors that might affect both choice of partner and cardiovascular risk factors. These factors included demographics (age, household income, education) and anthropometric measures (body mass index [BMI], calculated as weight in kilograms divided by height in meters squared; waist circumference). To address the potential influence of these factors in the full study population, partial correlations were determined after sequential adjustment for (model I) age of both partners (as linear terms), (model II) household income (in 1000 yuan [US $156]) and education level (in years) of both partners, (model III) smoking status of the male partner (because only 6 women were smokers), and then either (model IV) BMI of both partners or (model V) waist circumference of both partners (adjustment for BMI or waist was performed because of the possibility that a tendency to choose a spouse with similar body habitus to one’s own could be a potential basis for spousal concordance of cardiovascular risk factors). We used frequency tables to evaluate the prevalence of spousal concordance of ideal or nonideal status of cardiovascular risk factors, with the respective thresholds for ideal status defined as follows: (1) systolic blood pressure less than 120 mm Hg; (2) diastolic blood pressure less than 80 mm Hg; (3) total cholesterol less than or equal to 5.17 mmol/L; (4) LDL cholesterol less than or equal to 3.5 mmol/L; (5) HDL cholesterol greater than or equal to 1.3 mmol/L; and (6) triglycerides less than or equal to 1.7 mmol/L.^11^ For each risk factor, we then used logistic regression to produce odds ratios to measure the strength of association between spousal nonideal status and the direction of association. Lastly, Spearman correlations of cardiovascular risk factors between spouses were determined in the 45 couples in which the woman subsequently had a pregnancy complicated by gestational diabetes, preeclampsia, or preterm delivery (ie, pregnancy outcomes that are associated with future maternal risk of CVD).^12,13,14^ All tests were 2-sided and performed at level of significance of *P* < .05.

## Results

Table 1 shows the demographic and clinical characteristics of the women and men composing the 831 participating couples. The participants were young adults, with a mean (SD) age of 24 (3) years among the women and 26 (4) years among the men. Mean (SD) BMI was 20.2 (2.5) among the women and 22.0 (2.6) among the men. Although only 6 women (0.8%) reported current smoking, 327 men (42.7%) reported current smoking. Despite this marked difference in smoking, the Figure shows that there were significant correlations between spouses in systolic blood pressure (*r* = 0.43; *P* < .001), diastolic blood pressure (*r* = 0.36; *P* < .001), total cholesterol (*r* = 0.13; *P* < .001), LDL cholesterol (*r* = 0.11; *P* = .003), HDL cholesterol (*r* = 0.22; *P* < .001), and triglycerides (*r* = 0.13; *P* < .001).

**Table 1.** Demographics and Cardiovascular Risk Factors in Newly Married Couples

Characteristic	Mean (SD)	
	Women (n = 831)	Men (n = 831)
Age, y	24 (3)	26 (4)
Education, median (IQR), y^a^	9 (10-12)	9 (9-12)
Household income, median (IQR), ¥1000^a^	20 (13-30)	20 (13-30)
Current smoking, No. (%)	6 (0.8)	327 (42.7)
BMI	20.2 (2.5)	22.0 (2.6)
Waist circumference, cm	70.5 (7.5)	75.4 (7.9)
Blood pressure, mm Hg		
Systolic	110 (12)	119 (12)
Diastolic	71 (9)	76 (9)
Cholesterol, mmol/L		
Total	3.8 (1.1)	4.3 (0.9)
LDL	2.1 (0.8)	2.4 (0.7)
HDL	1.5 (0.4)	1.2 (0.3)
Triglycerides, median (IQR), mmol/L^a^	0.9 (0.6-1.3)	1.3 (0.9-1.9)

Abbreviations: BMI, body mass index (calculated as weight in kilograms divided by height in meters squared); HDL, high-density lipoprotein; LDL, low-density lipoprotein.

^a^ Continuous variables with skewed distribution.

**Figure.**
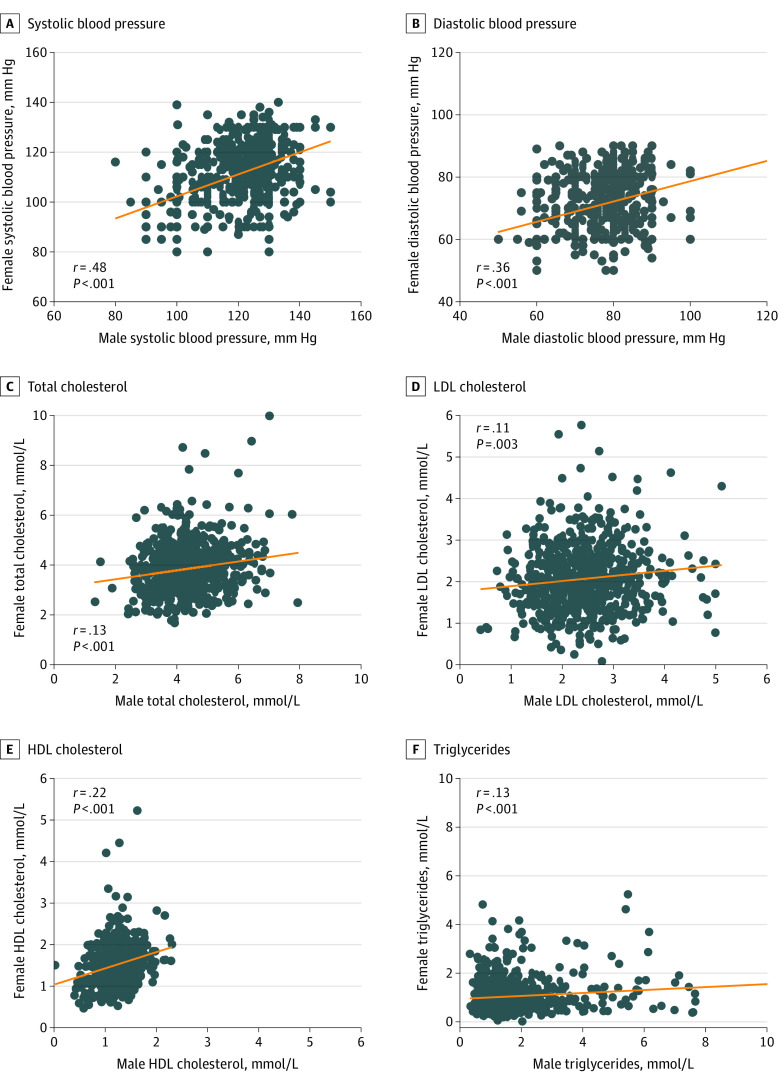
Spousal Correlations of Cardiovascular Risk Factors The figure shows spousal correlations of cardiovascular risk factors for systolic blood pressure (A), diastolic blood pressure (B), total cholesterol (C), LDL cholesterol (D), HDL cholesterol (E), and triglycerides (F).

To evaluate the potential association of smoking with the spousal concordance of cardiovascular risk factors, we first stratified the couples into those in which neither partner smoked (n = 428) and those in which at least 1 partner smoked (n = 332) (there were 71 other couples in which smoking status of both partners could not be confirmed). As shown in Table 2, all of the spousal correlations for blood pressure and lipids observed in the Figure were maintained in the nonsmoking couples. In couples where at least 1 partner smoked, these significant associations were maintained for systolic blood pressure (*r* = 0.48; *P* < .001), diastolic blood pressure (*r* = 0.41; *P* < .001), HDL cholesterol (*r* = 0.15; *P* = .006), and triglycerides (*r* = 0.11; *P* = .04). Of note, however, the significant spousal associations of total cholesterol (*r* = 0.20; *P* < .001) and LDL (*r* = 0.16; *P* = .001) in nonsmoking couples stood in stark contrast with the absence thereof in couples where at least 1 partner smoked (total cholesterol: *r* = 0.01; *P* = .89; LDL cholesterol: *r* = 0.02; *P* = .71), likely reflecting the asymmetric role of smoking in the men.

**Table 2.** Spearman Correlations of Cardiovascular Risk Factors Between Women and Their Partners According to Smoking Status

Characteristic	Couples where neither partner smokes (n = 428)		Couples where at least 1 partner smokes (n = 332)	
	*r* (95% CI)	*P* value	*r* (95% CI)	*P* value
Blood pressure				
Systolic	0.38 (0.30 to 0.46)	<.001	0.48 (0.40 to 0.56)	<.001
Diastolic	0.33 (0.24 to 0.41)	<.001	0.41 (0.31 to 0.50)	<.001
Cholesterol				
Total	0.20 (0.10 to 0.29)	<.001	0.01 (−0.10 to 0.11)	.89
LDL	0.16 (0.06 to 0.25)	.001	0.02 (−0.09 to 0.13)	.71
HDL	0.27 (0.18 to 0.36)	<.001	0.15 (0.04 to 0.25)	.006
Triglycerides	0.14 (0.04 to 0.23)	.005	0.11 (0.004 to 0.22)	.04

Abbreviations: BMI, body mass index (calculated as weight in kilograms divided by height in meters squared); HDL, high-density lipoprotein; LDL, low-density lipoprotein.

There were 71 couples in which smoking status in both partners was not available. These couples showed no significant differences from the included 760 couples in age, education, household income, BMI, or waist circumference for both the women and the men, respectively (data not shown).

We next evaluated adjustment of demographic variables (age, income, education) and anthropometric measures (BMI, waist circumference) on the spousal correlations of cardiovascular risk factors in the complete study population (Table 3). Adjustment for the age of both partners (model I) yielded little change in the correlations. Further adjustment for household income, education level of both partners and smoking status of the male partner ameliorated the associations of total cholesterol and LDL (model II and model III). Finally, further adjustment for either BMI of both partners (model IV) or waist circumference of both partners (model V) yielded little change in the significant spousal correlations of systolic blood pressure (*r* = 0.42; *P* < .001), diastolic blood pressure (*r* = 0.34; *P* < .001), HDL cholesterol (*r* = 0.17; *P* < .001), and triglycerides (*r* = 0.10; *P* = .04).

**Table 3.** Adjusted Spearman Correlations of Cardiovascular Risk Factors Between Women and Their Partners

	r (95% CI)					
	Unadjusted	Model I	Model II	Model III	Model IV	Model V
Age	0.56 (0.51 to 0.61)	NA	NA	NA	NA	NA
Education	0.39 (0.33 to 0.45)	0.39 (0.32 to 0.44)	NA	NA	NA	NA
BMI	0.10 (0.02 to 0.18)	0.08 (−0.009 to 0.16)	0.06 (−0.03 to 0.15)	0.07 (−0.02 to 0.17)	NA	0.02 (−0.08 to 0.12)
Waist	0.14 (0.06 to 0.23)	0.13 (0.05 to 0.22)	0.13 (0.04 to 0.22)	0.13 (0.04 to 0.22)	0.12 (0.03 to 0.21)	NA
Blood pressure						
Systolic	0.43 (0.37 to 0.48)	0.42 (0.36 to 0.48)	0.42 (0.34 to 0.49)	0.41 (0.34 to 0.48)	0.43 (0.35 to 0.50)	0.42 (0.34 to 0.49)
Diastolic	0.36 (0.30 to 0.42)	0.36 (0.29 to 0.42)	0.34 (0.26 to 0.42)	0.34 (0.26 to 0.42)	0.34 (0.26 to 0.42)	0.34 (0.26 to 0.42)
Cholesterol						
Total	0.13 (0.06 to 0.19)	0.11 (0.04 to 0.18)	0.10 (0.01 to 0.18)	0.08 (−0.002 to 0.17)	0.09 (0.005 to 0.18)	0.09 (−0.003 to 0.18)
LDL	0.11 (0.04 to 0.17)	0.09 (0.01 to 0.16)	0.07 (−0.02 to 0.16)	0.07 (−0.02 to 0.16)	0.08 (−0.01 to 0.17)	0.08 (−0.01 to 0.17)
HDL	0.22 (0.16 to 0.29)	0.22 (0.15 to 0.29)	0.16 (0.07 to 0.24)	0.16 (0.07 to 0.24)	0.17 (0.09 to 0.26)	0.17 (0.08 to 0.25)
Triglycerides	0.13 (0.06 to 0.20)	0.14 (0.07 to 0.21)	0.09 (0.0002 to 0.17)	0.09 (−0.001 to 0.17)	0.10 (0.007 to 0.18)	0.10 (0.006 to 0.18)

Abbreviations: BMI, body mass index (calculated as weight in kilograms divided by height in meters squared); HDL, high-density lipoprotein; LDL, low-density lipoprotein; NA, not applicable.

Spearman correlations of cardiovascular risk factors between women and their partner, unadjusted and after adjustment as follows: Model I: Adjusted for age of both partners. Model II: Adjusted for Model I covariates + household income and education of both partners. Model III: Adjusted for Model II covariates + smoking status of male partner. Model IV: Adjusted for Model III covariates + BMI of both partners. Model V: Adjusted for Model III covariates + waist circumference of both partners.

We next evaluated spousal concordance of ideal or nonideal status of cardiovascular risk factors (Table 4). This approach showed that the spousal concordance of ideal or nonideal status was greater than 50% for each risk factor, suggesting that spousal unhealthy outcomes might be associated. Of note, a woman with nonideal systolic blood pressure was 3 times more likely to have a male partner with nonideal systolic blood pressure (odds ratio [OR], 3.09; 95% CI, 2.17-4.41). Similarly, a woman with nonideal diastolic blood pressure or HDL cholesterol was more likely to have a male partner with nonideal diastolic blood pressure (OR, 2.48; 95% CI, 1.74-3.53) or nonideal HDL cholesterol (OR, 2.11; 95% CI, 1.53-2.90), respectively. For the other cardiovascular risk factors, there was no significant association of categorical outcomes between the female and male partners.

**Table 4.** Spousal Concordance of Ideal or Nonideal Status of Cardiovascular Risk Factors

Characteristic	Ideal status	%			Odds ratio (95% CI)
		Concordant		Discordant: 1 spouse ideal	
		Both ideal	Both nonideal		
Blood pressure					
Systolic	<120 mm Hg	21.4	36.3	42.3	3.09 (2.17-4.41)
Diastolic	<80 mm Hg	14.8	45.2	40.1	2.48 (1.74-3.53)
Cholesterol					
Total	≤5.17 mmol/l	1.6	78.1	20.3	1.56 (0.82-2.99)
LDL	≤3.5 mmol/l	0.5	90.0	9.5	2.23 (0.75-6.65)
HDL	≥1.3 mmol/l	22.5	30.7	46.8	2.11 (1.53-2.90)
Triglycerides	≤1.7 mmol/l	4.5	60.3	35.2	1.42 (0.91-2.22)

Abbreviations: HDL, high-density lipoprotein; LDL, low-density lipoprotein.

Among the 831 newly married couples, 738 went on to have a subsequent pregnancy, during which 45 women developed either gestational diabetes, preeclampsia, or preterm delivery. Because each of these pregnancy outcomes identifies future maternal risk of CVD such that postpartum surveillance of vascular risk factors has been recommended in affected women,^14^ we sought to evaluate spousal concordance in these couples (to see whether concomitant postpartum assessment of the male partners might also warrant consideration). In these 45 couples, there were indeed significant spousal associations of pregravid systolic blood pressure (*r* = 0.51; *P* < .001) and HDL cholesterol (*r* = 0.50; *P* < .001) (eFigure in the Supplement). It thus emerges that the recommended postpartum surveillance of women with these pregnancy outcomes could provide an opportunity for couple-based care that may identify male partners with an adverse cardiovascular risk factor profile.

## Discussion

Although many previous studies have shown concordance of cardiovascular risk factors between older and middle-aged marital partners, it has remained unclear whether the basis underlying this phenomenon is their shared home environment and lifestyle or assortative mating.^1,2,3,4,5,6,7^ To address this question, previous studies have taken 1 of 3 indirect approaches to either support or refute assortative mating. First, the observation that spousal concordance of blood pressure, lipids, and anthropometrics does not vary with age of the individuals has been forwarded as supportive evidence of assortative mating.^15^ Second, it has been reported that spousal correlations of blood pressure and lipids showed inconsistent trends in relation to the duration of marriage in couples that were married between 1945 and 1964.^15^ The authors thus concluded that the lack of consistent trends in the impact of duration of marriage was more supportive of assortative mating than of the shared home environment.^16^ Third, other researchers have argued that BMI can provide a surrogate for assortative mating.^7,8^ Accordingly, when spousal correlations were not decreased upon adjustment for BMI, they have suggested that assortative mating may not be the basis of the associations.

In the current study, we have taken a more direct approach by studying young, newly married couples in whom the impact of the shared home environment on spousal correlations has likely not yet emerged. With this approach, we found clear correlations in cardiovascular risk factors between newly married partners, with 3 additional points to note. First, the correlations for blood pressure were stronger than those for lipid measures. Indeed, the spousal correlations for both systolic and diastolic blood pressure were also higher than those estimated from meta-analyses of 30 studies and 27 studies, respectively.^5^ It is possible that these differences may reflect the lack of other potentially confounding influences in this young healthy population (as compared with the older populations of previous studies in whom comorbidities and medications may be more likely to exert an asymmetric effect on blood pressure in 1 partner and not the other). Second, as shown in Table 3, adjustment for either BMI or waist circumference yielded very little change in the correlations for both blood pressure and lipid measures. This finding argues against the concept that the underlying factors by which assortative mating may drive spousal correlations necessarily converge through body habitus. Third, although smoking status has been the most concordant cardiovascular risk factor within couples in previous meta-analyses,^5^ our population was different in this regard, with high rates of smoking in men (42.7%) but very low rates in women (0.8%). In this setting, the stratified analyses in Table 2 show that spousal correlations are present for blood pressure and all of the lipid measures in the nonsmoking couples and the asymmetric impact of smoking in the men served to ameliorate the associations for total and LDL cholesterol (but not blood pressure, HDL cholesterol, or triglycerides) in the couples with discordant smoking status.

### Implications

Our findings may hold implications for preventive care strategies that are directed toward young people, with the goal of reducing the future incidence of CVD. Specifically, the concordance of blood pressure and lipids between newlywed partners in their twenties raises the possibility that the identification of a young person with an adverse cardiovascular risk factor profile may warrant the suggestion of their partner undergoing evaluation as well. One of the challenges for such preventive care is that young people rarely seek routine clinical surveillance. In this context, pregnancy provides a unique opportunity wherein young women typically engage with the health care system and may be particularly receptive to making healthy behavioral changes. The current findings (and their support of the concept of assortative mating) suggest that the clinical detection of at-risk women may also provide an opportunity to identify their at-risk partners. Indeed, our demonstration of robust spousal concordance of blood pressure and HDL cholesterol in couples where the women subsequently developed gestational diabetes, preeclampsia, or preterm delivery serves to highlight this opportunity. These pregnancy outcomes are associated with both an adverse cardiovascular factor profile and an elevated future risk of CVD.^12,13,14,17^ As such, it is recommended that women with these pregnancy outcomes undergo postpartum assessment of cardiovascular risk factors.^14^ The current findings raise the possibility that family-based assessment may instead offer a broader opportunity for preventive care in both parents at an early age. Further study of this possibility is warranted.

### Limitations

A limitation of this study is that the cross-sectional assessment of the male partners precludes evaluation of changes in spousal associations over time. In addition, we did not collect data on physical activity and diet, which might provide insight into some of the lifestyle practices in which the newly married couples may have found mutual compatibility (as per assortative mating). Because this study was performed in a single region (Liuyang, China), studies in other settings are needed to determine if these findings are generalizable to other populations. Another limitation is that the duration of cohabitation was not specifically ascertained. That said, this duration was likely modest in this young, newly married patient population in a setting where cohabitation prior to marriage is generally not common. Accordingly, the observed correlations are more likely to reflect assortative mating than the impact of many years of a shared environment and lifestyle.

## Conclusions

This cohort study found that there is spousal concordance of cardiovascular risk factors in young, newly married couples. These associations are suggestive of assortative mating as a basis for similar observations in older couples. Moreover, they are not dependent on age, socioeconomics, smoking, or body habitus. These findings in newly married spouses may support the consideration of couple-based care when young adults are found to have risk factors for the future development of CVD.

## Notes

Supplement. **eFigure.** Plots Showing the Spousal Correlations of (A) Systolic Blood Pressure, (B) Diastolic Blood Pressure, (C) Total Cholesterol, (D) LDL Cholesterol, (E) HDL Cholesterol, and (F) Triglycerides, Within the 45 Couples in Which the Woman Subsequently Had Gestational Diabetes, Pre-eclampsia or Preterm Delivery
