# Supplementary material for: Spousal Concordance of Cardiovascular Risk Factors in Newly Married Couples in China
Source: JAMA Netw Open. 2021 Dec 22;4(12):e2140578. doi: 10.1001/jamanetworkopen.2021.40578 (PMC8696567; doi:10.1001/jamanetworkopen.2021.40578)
Supplement: Supplement. — eFigure. Plots Showing the Spousal Correlations of (A) Systolic Blood Pressure, (B) Diastolic Blood Pressure, (C) Total Cholesterol, (D) LDL Cholesterol, (E) HDL Cholesterol, and (F) Triglycerides, Within the 45 Couples in Which the Woman Subsequently Had Gestational Diabetes, Pre-eclampsia or Preterm Delivery [file jamanetwopen-e2140578-s001.pdf]

Supplemental Online Content

Retnakaran R, Wen SW, Tan H, et al. Spousal concordance of cardiovascular risk factors in newly married couples in China. *JAMA Netw Open.* 2021;4(12):e2140578. doi:10.1001/jamanetworkopen.2021.40578

eFigure. Plots Showing the Spousal Correlations of (A) Systolic Blood Pressure, (B) Diastolic Blood Pressure, (C) Total Cholesterol, (D) LDL Cholesterol, (E) HDL Cholesterol, and (F) Triglycerides, Within the 45 Couples in Which the Woman Subsequently Had Gestational Diabetes, Pre-eclampsia or Preterm Delivery

This supplemental material has been provided by the authors to give readers additional information about their work.

© 2021 Retnakaran R et al. *JAMA Network Open*.

eFigure: Plots showing the spousal correlations of (A) systolic blood pressure, (B) diastolic blood pressure, (C) total cholesterol, (D) LDL cholesterol, (E) HDL cholesterol, and (F) triglycerides, within the 45 couples in which the woman subsequently had gestational diabetes, pre-eclampsia or preterm delivery

Panel A : Systolic blood pressure

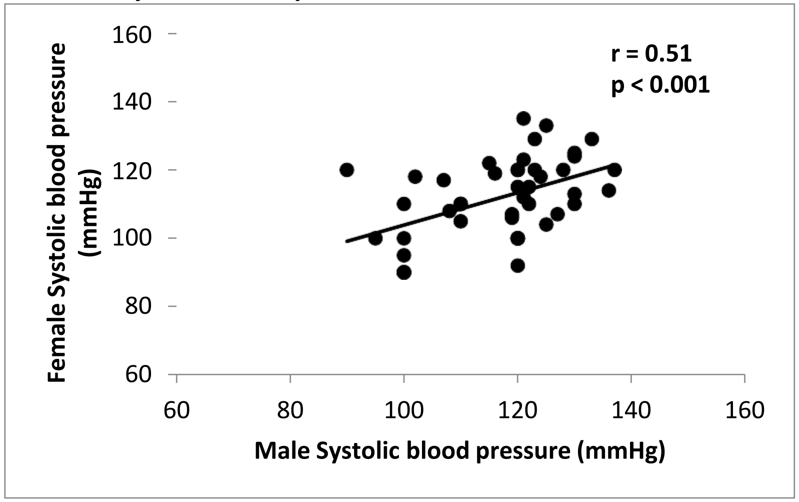

Panel B: Diastolic blood pressure

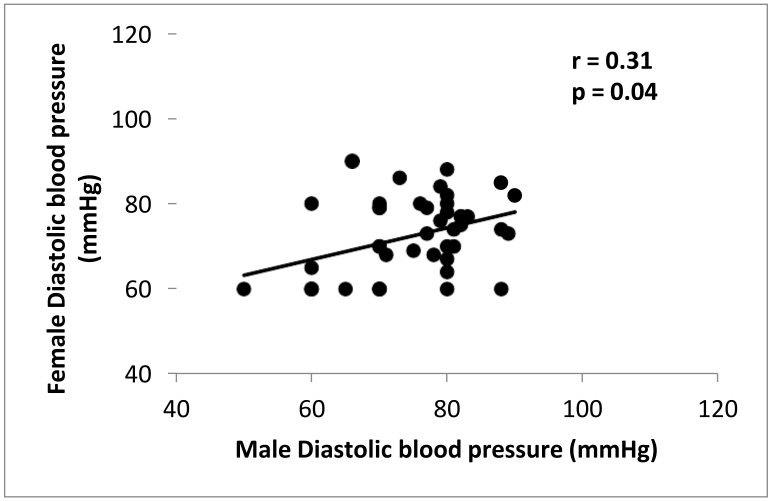

Panel C: Total cholesterol

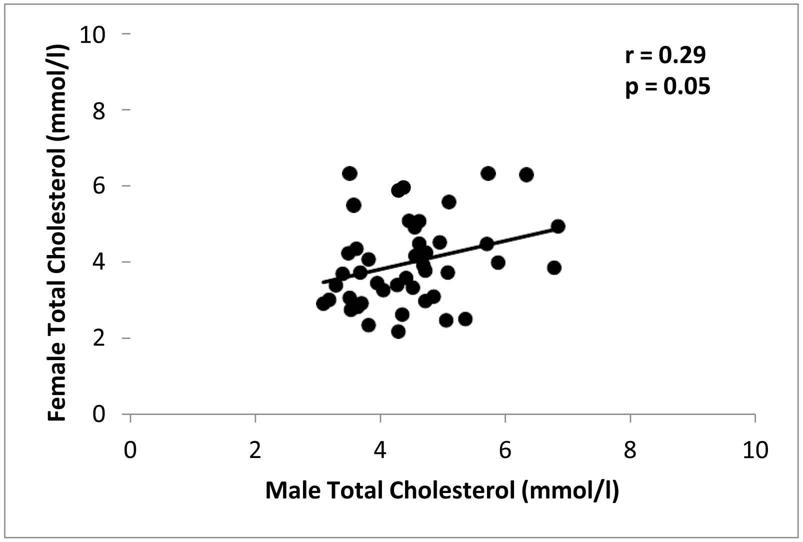

Panel D: LDL cholesterol

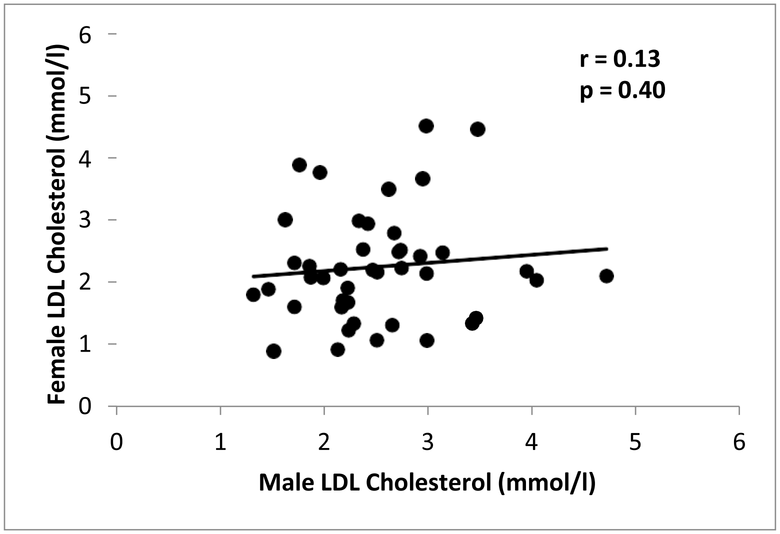

© 2021 Retnakaran R et al. *JAMA Network Open*.

Panel E: HDL cholesterol

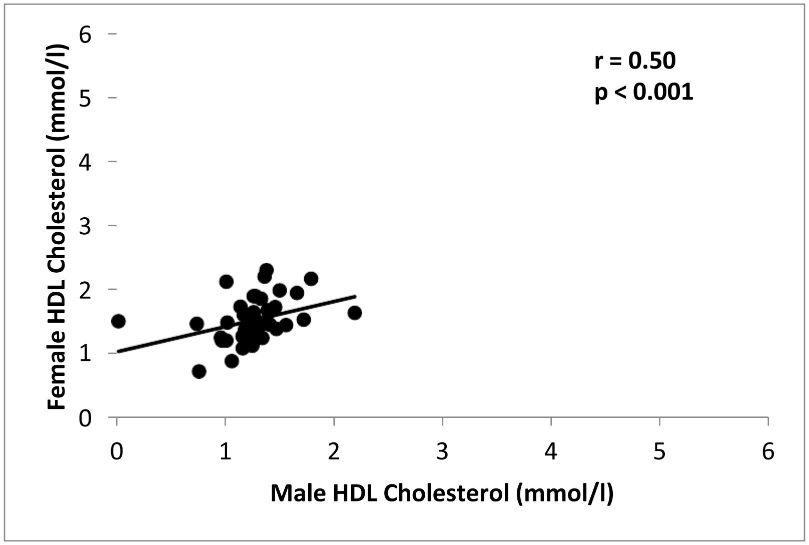

Panel F: Triglycerides

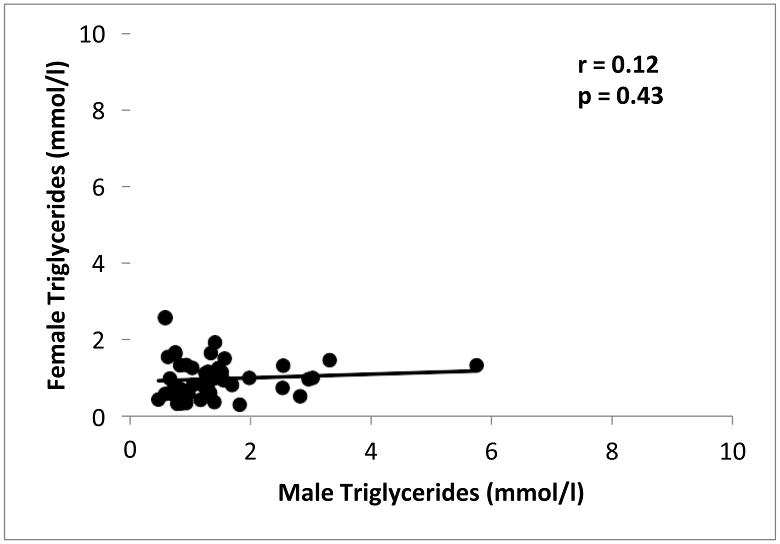

© 2021 Retnakaran R et al. *JAMA Network Open*.
